# Epigenetics and Heart Development

**DOI:** 10.3389/fcell.2021.637996

**Published:** 2021-05-06

**Authors:** Rajani M. George, Anthony B. Firulli

**Affiliations:** Herman B Wells Center for Pediatric Research Department of Pediatrics, Anatomy, Biochemistry, and Medical and Molecular Genetics, Indiana University School of Medicine, Indianapolis, IN, United States

**Keywords:** epigenetics, cardiac development, CTCF, TADs, heart

## Abstract

Epigenetic control of gene expression during cardiac development and disease has been a topic of intense research in recent years. Advances in experimental methods to study DNA accessibility, transcription factor occupancy, and chromatin conformation capture technologies have helped identify regions of chromatin structure that play a role in regulating access of transcription factors to the promoter elements of genes, thereby modulating expression. These chromatin structures facilitate enhancer contacts across large genomic distances and function to insulate genes from *cis*-regulatory elements that lie outside the boundaries for the gene of interest. Changes in transcription factor occupancy due to changes in chromatin accessibility have been implicated in congenital heart disease. However, the factors controlling this process and their role in changing gene expression during development or disease remain unclear. In this review, we focus on recent advances in the understanding of epigenetic factors controlling cardiac morphogenesis and their role in diseases.

## Introduction

The heart is the first functional organ to develop during embryogenesis (Christoffels and Jensen, 2020). The earliest cardiac progenitors are specified from the emerging mesoderm during gastrulation and form the cardiac crescent. Cardiac progenitor cells (CPCs) within the cardiac crescent come together and are distinguished into two populations of progenitor cells: the primary and secondary heart fields (Kelly et al., 2014). CPCs expressing transcription factors (TFs) *Nkx2-5*, *Gata4*, and *Tbx5* coalesce at midline and form a linear tube that circulates blood in the developing embryo (Bruneau, 2013). As cells within the primary heart field proliferate to form the left ventricle, progenitor cells from the second heart field, expressing the markers *Isl1* and *Tbx1*, move into the developing heart at the arterial and venous poles and eventually give rise to the right ventricle, outflow tract, and the majority of the interventricular septum (Black, 2007). Differential addition of CPCs from the two poles causes the heart to undergo rightward looping, which juxtaposes the atria and ventricles in their final orientation, and through septation, the heart forms its final four-chambered structure (Sylva et al., 2014). The adult mammalian heart is composed of a multitude of cell types: atrial and ventricular cardiomyocytes (CMs) make up most of the heart volume; endocardial cells line the chambers, the lumen of arteries, and specialized valve cells; smooth muscle cells form the aorta and coronary arteries; the epicardium sheaths the heart; and specialized cells form the cardiac conduction system that drives chamber contraction (Pinto et al., 2016).

Cardiac development requires finely tuned gene expression within these various cell types (Olson, 2006). Gene expression is modulated by TFs that bind to DNA regulatory elements to activate or repress transcription. TFs need to have access to consensus DNA binding sequences to recruit the transcriptional machinery, which then initiates transcription. DNA within the nucleus is highly organized into chromatin, a complex of DNA and proteins. Chromatin organization is dynamic and undergoes changes in its structure from loose and open to tightly condensed and closed. Conformational changes to chromatin modulate gene expression by controlling the TFs’ accessibility to the DNA binding sites (Voss and Hager, 2014). Epigenetic modifications do not alter the DNA sequence itself but can change the chromatin structure to modulate its accessibility (Allfrey et al., 1964). The need to expand our understanding of the role that epigenetic regulation plays in gene expression has led scientists to study the structure of the three-dimensional (3D) genome and the hierarchy of chromatin organization (Yu and Ren, 2017). The study of the epigenome has led to the characterization of DNA base methylation, posttranslational histone modifications, the interactions of long non-coding RNA molecules, and changes in chromatin folding. These epigenetic events allow (or block) *trans*-acting factors to interact with specific *cis-*elements located within transcriptional enhancers, thus fine-tuning gene expression during development. Studies examining epigenetic changes have given new insight into gene expression changes that take place during disease conditions such as dilated cardiomyopathy. Recent work has also shed more light on diseases referred to as cohesinopathies, which are caused by disruptions to cohesin, a protein complex that helps form the 3D chromatin structure. In this review, we give an overview of the various mechanisms of epigenetic regulation and recent developments examining regulatory mechanisms in the context of cardiac development and disease.

## Mechanisms of Epigenetic Regulation of Gene Expression

### DNA Methylation

DNA methylation is an early epigenomic change, occurring during DNA replication to mark the daughter strand (Riggs, 1975; Bird, 1978). Methylation of DNA, specifically at the fifth carbon of the cytosine base, occurs on CpG dinucleotides and, in the context of epigenetic regulation, most commonly leads to transcriptional repression (Greenberg and Bourc’his, 2019). Analysis of DNA methylation status during mouse cardiac development (comparing E11.5 to E14.5) shows >50% change in methylation status within a subset of genes involved in heart development and cardiac tissue growth (Chamberlain et al., 2014). When *Dnmt3b* (DNA methyltransferase 3b), an enzyme that catalyzes transfer of methyl groups to CpG motifs, is deleted within the endocardium using *Nfatc1*^*Cre*^ (Wu et al., 2011), qRT-PCR analysis at E11.5 and E14.5 reveals significantly increased levels of *Has3* (*hyaluronan synthase 3*). *Has3* is an extracellular matrix remodeling enzyme and, in the context of heart development, is required for endothelial-to-mesenchymal transition and valve formation, therefore suggesting a link between the HAS3 methylation status and regulation during cardiac development (Chamberlain et al., 2014). Knockdown of another member of the same family of DNA methyltransferases, *Dnmt3a*, using siRNA in mouse embryonic CMs resulted in an observed decrease in beating frequency, defective contractile movement, and disrupted sarcomere assembly (Fang et al., 2016). RNA-seq and methylome analyses identified increased expression and associated decreased CpG methylation at promoters of the following CM structural genes: *Myh7*, *Myh7b*, *Tnni3*, and *Tnnt2* (Fang et al., 2016). The importance of CpG methylation in regulating the switch between fetal and adult CM gene expression program is further illustrated by the conditional deletion of *Dnmt3a/b* in CMs using the *Mlc2a* promoter to drive *Cre recombinase* expression (Gilsbach et al., 2014). MethylC-seq analysis in these mutant CMs reveal reduced postnatal *de novo* methylation of fetal *Troponin1* (*Tnni1*), which partially relieves repression of *Tnni1* (Gilsbach et al., 2014). CpG methylation and gene expression analysis of purified embryonic human CMs from fetal (16–24 weeks of pregnancy), infant (1–12 months), and adult (46–60 years) patient samples show dynamic changes in CpG profile and genomic regions that exhibit low levels of CpG-marked enhancers or silencers that lie in *cis* with genes involved in CM maturation, reflecting the change in their mitotic ability through development (Gilsbach et al., 2018). Work by Li et al. (2016) examining another family of CpG modifiers, *Tet1/2/3*, which facilitates demethylation by oxidizing CpG residues, in mouse embryonic stem cells (mESCs) shows that loss of *Tet3* alone or *Tet1*, *Tet2*, and *Tet3* triple knockout leads to improper adoption of cardiac mesodermal fate at the expense of neural cell fate as determined by qRT-PCR analysis of neuronal markers *Sox1* and *Foxg1* and CM markers *Nkx2-5*, *Myh6*, *Myh7*, and *Tnnt2*.

In human disease conditions, variability in methylome status is observed (Movassagh et al., 2011; Haas et al., 2013). Using the Illumina 450K methylation assay on tissues from dilated cardiomyopathy patients reveals significant changes in the methylation status of cardiac disease-associated genes such as *NPPA* and *NPPB*, with methylation status validation by MassARRAY (Meder et al., 2017). Analysis of non-failing donor human hearts and cardiac patients for DNA methylation signature using array-based Illumina Infinium HumanMethylation450 BeadChips reveals 168 differentially methylated CpG loci in atrial and ventricular heart tissues, with 24 of these loci in predicted human heart-specific enhancers (Hoff et al., 2019). In human patients with ischemic cardiomyopathy due to coronary heart disease, methylome analysis using Illumina Infinium HumanMethylation450 BeadChips shows significant increase in CpG methylation and transcriptional repression of the citric acid (TCA) cycle and respiratory electron transport gene network (Pepin et al., 2019). Authors identify KLF15, a Krüppel-like factor, as a target for EZH2 that facilitates a metabolic reprogramming as shown by increased CpG methylation, EZH2 binding at the KLF15 promoter, and KLF15-mediated suppression of key oxidative metabolic genes (Pepin et al., 2019). Thus, CpG methylation is established as a key epigenetic signature in CMs that distinguishes their gene expression and function including an important role for CpG methylation in the metabolic switch that occurs in human heart CMs during failure.

### Chromatin Remodelers

The 2 m of naked DNA found inside a 5-μm mammalian nucleus would be highly suspectable to breaks and damage if it was not packaged into an organized structure that allows for reliable replication, transcription, and repair (Felsenfeld and Groudine, 2003). Chromatin is a complex of negatively charged DNA, which weakly interacts with positively charged residues found in histone proteins that allow for the tight and safe packaging of genetic information within the nucleus (McGhee and Felsenfeld, 1980). The functional unit of chromatin is the nucleosome, which is formed by a core of histone proteins H2A, H2B, H3, and H4, around which DNAs (147 base pairs) are wrapped (Kornberg, 1974). These core histone proteins are subject to various posttranslational modifications, including acetylation, methylation, phosphorylation, and ubiquitination at specific residues within the amino-terminal histone tails or within the globular/core domains of histones (Clapier and Cairns, 2009). Various combinations of these posttranslational modifications make up the epigenetic code or histone code that marks regions of open or closed chromatin (Karlic et al., 2010). Transcriptionally active (open) regions of chromatin contain high levels of histone 3 (H3) monoacetylated (ac) at Lys-9 and Lys-14 (H3K9ac and H3K14ac). Trimethylated (me3) Lys-4 (H3K4me3) is present within promoter regions. Other modifications, such as dimethylated (me2) Lys-79 (H3K79me2) and trimethylated Lys-36 (H3K36me3), mark transcriptionally active coding regions. Repressive epigenetic signals are regions of deacetylation and histone H3 trimethylation of Lys-9 (H3K9me3) and Lys-27 (H3K27me3) (Barski et al., 2007; Ernst et al., 2011). Enhancer sequences (evolutionarily conserved non-coding regions of DNA that TFs bind to) are required to drive gene expression. These enhancer regions exhibit unique epigenetic signals as well as increased H3K27me3 and lack of H3K27ac, which poises them for gene activation. The H3K27ac modification is indicative of active enhancers (Rada-Iglesias et al., 2011). These epigenetic marks have been employed to help identify novel enhancers involved in cardiac development (Nord et al., 2013).

Histone marks alone are not always a predictor for enhancer activity; a comparison of chromatin immunoprecipitation using next-generation sequencing (ChIP-seq) profiles of cardiac-specific TFs (GATA4, MEF2A, MEF2C, NKX2-5, SRF, TBX5, and TEAD1) showed that only 16% of regions with TF occupancy overlapped with H3K27ac chromatin marks within fetal cardiac tissues (Akerberg et al., 2019). Analysis of chromatin accessibility using ATAC-seq (Assay for Transposase-Accessible Chromatin using sequencing) revealed that multiple TF binding regions strongly corelated with the ATAC-seq signal; 100% of regions binding all seven TFs have a strong ATAC-seq signal (Akerberg et al., 2019). These genome-wide screening experiments examining chromatin accessibility and the associated histone code in CMs show the importance of epigenetic remodeling, driving TF access to reprogram the CM transcriptome during the transition from fetal to adult stages. Experiments seeking to correlate the differences in the distribution histone modifications and gene expression were performed by first subjecting adult mice to transverse aortic constriction inducing cardiac hypertrophy and then subsequently heart failure, followed by screening of the diseased hearts for changes in seven different histone modifications and gene expression (Papait et al., 2013). Of 1,109 differentially regulated genes, 596 exhibit at least one altered histone modification at their promoter and 325 genes observed an upregulation or downregulation of gene expression coincident with the histone change (Papait et al., 2013).

A well-studied epigenetic modifier during heart development, EP300, is a histone acetyltransferase (HAT) that acetylates H3 on lysine 27 (H3K27ac) and binds within the promoter regions of a number of critical heart genes such as *Gata4*, *Nkx2-5*, and *Mef2c* to activate the transcription of these cardiac TFs (Takaya et al., 2008; Sun et al., 2010; Trivedi et al., 2010). Indeed, a point mutation in EP300 leads to atrial septal defects and ventricular septal defects (Shikama et al., 2003). Conditional inactivation of a subunit (*Ezh2*) of PRC2, which establishes the chromatin mark H3K27me3, using *Nkx2-5^*Cre*^* (*Ezh2^*fl/fl*^ Nkx2-5^*Cre*^*) causes perinatal lethality with only 2% of pups surviving to postnatal day 20 (He et al., 2012). In this study, no significant apoptosis is detected via terminal deoxynucleotidyl transferase dUTP nick end labeling (TUNEL); however, CM proliferation as measured by phosphorylated histone H3 immunohistochemistry is twofold reduced in mutant CMs at E16.5. Transcriptomic analysis via qRT-PCR and RNA-seq indicates that *Pax6*, *Isl1*, and *Six1*, genes that are expressed in early cardiac progenitors and downregulated in differentiated CMs, are significantly overexpressed in E12.5 mutant ventricles, suggesting that effective cardiac progenitor differentiation to CMs requires repressive H3K27me3 activity (He et al., 2012).

Another class of proteins regulate chromatin by non-covalent enzymatic activity. The SWI/SNF complexes consist of a core ATPase that utilizes energy from ATP hydrolysis to modify chromatin by changing nucleosome DNA contacts, moving nucleosomes along the DNA, and removing or exchanging the DNA from nucleosomes (Ho and Crabtree, 2010; Hargreaves and Crabtree, 2011). Early cardiac development requires finely tuned epigenetic regulation by these complexes as demonstrated by ectopic cardiogenesis when BAF60C (SMARCD3), a cardiac-specific ATP-dependent chromatin remodeling protein, and GATA4 and TBX5 are expressed in the non-cardiogenic mouse embryo mesoderm (Lickert et al., 2004; Takeuchi and Bruneau, 2009). RNA-seq analysis of hearts of mice, where *Baf60c* is conditionally knocked out from CMs using *Myh6-Cre*, showed mis-regulation of structural CM proteins, not TFs involved in cardiac development, possibly mediated by the direct interaction between myocardin (MYOCD) and BAF60C (Sun et al., 2018). Expression of the core ATPase subunit of the SWI/SNF complex, *Brg1*, is required in CMs for their maturation, as determined by isoform switching of *αMHC* and *βMHC* in the mouse myocardium (Hang et al., 2010), as well as in the endocardium to drive trabeculation (Stankunas et al., 2008). Knockout of *Arid2/BAF200* leads to embryonic lethality by E12.5–E14.5 due to improper myocardial development leading to thinning of ventricular walls and improper coronary formation (He et al., 2014). This family of chromatin remodelers is also implicated in disease, covered in further detail in section “Cardiac Diseases and the Epigenome”.

### Topologically Associating Domain (TAD)

**FIGURE 1:**
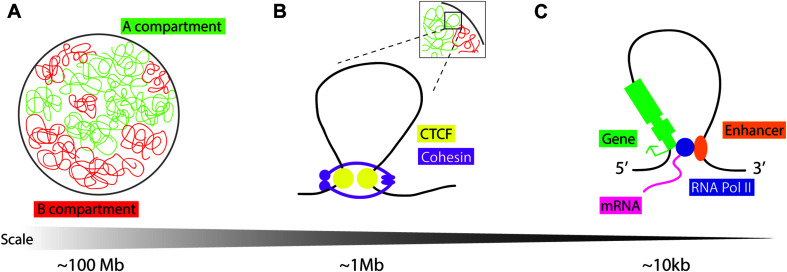
Schematic representation of chromatin loops. **(A)** Chromatin in the nucleus is distinguished into two compartments on the basis of histone modifications and transcriptional activity: compartment A is “open/active,” compartment B is “closed/repressed.” **(B)** Topologically associating domains (TADs) are dynamic chromatin structures that are extruded by cohesin, a protein complex consisting of SMC1, SMC3, RAD21, and STAG1/2. Loop extrusion is stabilized by the boundary molecule CTCF. **(C)** “Open” chromatin is permissive to loop formation to enable *cis*-enhancer binding to RNA Pol II and interaction with the gene promoter to facilitate tissue and temporally specific transcription.

Work published concurrently in 2012 used chromatin conformation capture in mammalian cells to identify large loops at the megabase scale: TADs (Dixon et al., 2012; Nora et al., 2012; Figure 1). TADs are not defined by a specific chromatin state alone but by the increased frequency of DNA interactions within a genomic region. Chromatin conformation capture experiments indirectly measure these contact frequencies at the whole-genome level using next-generation sequencing (Kempfer and Pombo, 2020). Live and single-molecule imaging at TADs shows that these structures are dynamic (Hansen et al., 2017). The generally accepted model for TAD formation is loop extrusion, where cohesin protein complexes are loaded and extrude chromatin into progressively larger loops until they either dissociate from chromatin, bump into each other, or run into insulator molecules such as the zinc finger CCCTC binding factor (CTCF) (Alipour and Marko, 2012; Sanborn et al., 2015; Fudenberg et al., 2016). These loops allow genomic sites that lie far apart in the linear genome to come into close spatial proximity to each other (Figure 1). Transcriptionally active and inactive TADs are organized in compartments A and B, respectively, which have different folding and compartmentalization configurations (Lieberman-Aiden et al., 2009; Wang et al., 2016). TAD compartment organization overlaps with histone modification: compartment A is enriched in markers for active chromatin (H3K27ac, H3K4me1/me3, H3K9me1, and the repressive H3K27me3 mark), while compartment B is enriched in the heterochromatin mark H3K9me3 (Rao et al., 2014). The mechanisms of TAD looping, their role in regulating gene expression, and their biological relevance remain unclear.

Disruption of TADs during development is implicated in ectopic gene expression and disease (Ibn-Salem et al., 2014; Lupianez et al., 2015, 2016; Muro et al., 2019). Comparison of genome architecture in primate, mouse, cattle, opossum, chicken, clawed frog, and zebrafish showed conservation of non-coding regions that coincide with TAD boundaries (Harmston et al., 2017; Krefting et al., 2018). However, the extent of TAD conservation across species and its functional significance are still unclear (Eres and Gilad, 2020). Although there is some variation in reports, mainly due to differences in resolution arising from experimental limitations and the types of computing tools used to generate models, the average size of a TAD is approximately 1 Mb (Dali and Blanchette, 2017; Zufferey et al., 2018).

CTCF protein is enriched at the TAD boundaries with pairs of CTCF DNA binding sites preferentially found in a convergent orientation, allowing the CTCF protein to act as a domain boundary molecule (Rao et al., 2014; Grubert et al., 2020; Figure 1). To study the function of CTCF in mediating TAD formation, researchers may employ an *in vitro* inducible knockout system, using the auxin-inducible degron (AID) tag with an eGFP cassette at the 3′ end of the CTCF coding sequence (Morawska and Ulrich, 2013). Cells are also transfected with *Tir1* F-box from *Oryza sativa*, which can bind to the AID tag in the presence of auxin, triggering targeted proteasome-dependent degradation of CTCF. Thus, adding auxin to cell culture media depletes CTCF protein to levels undetectable by western blot, and removal of auxin from growth media results in recovery of CTCF protein. The Bruneau lab targeted CTCF in mESCs by using this system, reporting that higher-order chromosome folding, i.e., compartments A and B, remain intact, as determined by Hi-C contact frequency mapping, with a limited effect on transcriptional state across the genome (Nora et al., 2017). Genes most affected by CTCF depletion tend to have enhancers/promoter regions in close proximity to CTCF sites. Reversal of CTCF depletion, by removal of auxin from growth media, leads to the re-formation of TADs (Nora et al., 2017), although the caveat to this result is that low levels of background CTCF protein can also facilitate TAD formation. More recently, the Bruneau lab has also uncovered the molecular basis for convergent CTCF motif orientation at TAD boundaries; the N-terminal portion of CTCF is responsible for its stabilization at one side of non-palindromic CTCF DNA binding sites during Cohesin-driven loop extrusion (Nora et al., 2020). *In vivo* knockout experiments have provided further information on the role of CTCF in TAD formation and maintenance.

In the mouse embryonic heart, conditional deletion of *CTCF* using *Nkx2-5^*Cre*^* leads to embryonic lethality by embryonic day (E)12.5 and myocardial thinning (Gomez-Velazquez et al., 2017). Conditional knockout embryos are phenotypically normal until E9.5, but by E10.5, the interventricular septum appears disorganized and progressively worsens in E11.5 embryos. The four chambers and atrioventricular canal form normally. No difference in apoptosis (TUNEL) or proliferation (phosphorylated histone H3) is observed. Transcriptomic analysis using RNA-seq of *Ctcf^*fl/fl*^;Nkx2-5^*Cre*^* embryos at E10.5 showed limited change in gene expression with genes being involved with mitochondrial function as the largest functional group showing differences (Gomez-Velazquez et al., 2017). Authors then go on to show changes in gene expression (*in situ* hybridization) and chromatin structure (4C-seq) at the *Iroquois* (*Irx*) gene cluster and adjoining genes, including a mitochondrial subunit gene *Ndufs6*. Mitochondria in *Ctcf^*fl/fl*^;Nkx2-5^*Cre*^* E11.5 CMs appear swollen and disorganized (Gomez-Velazquez et al., 2017). Work from the Vondriska lab employs a *α-Myosin Heavy chain* tamoxifen-inducible *Cre recombinase* (MerCreMer) mouse to generate *CTCF* conditional knockout (*CTCF-CKO*) in adult CMs (Rosa-Garrido et al., 2017). Depletion of CTCF levels after tamoxifen treatment led to a decrease in survivability, with impaired ejection fraction, left ventricular chamber dilation, and muscle hypertrophy at the organ and cell levels, with 100% mortality observed in conditional knockout mice 7 weeks after tamoxifen administration. Surprisingly, Hi-C analysis of hearts from these *CTCF-CKO* mice shows little change (<2%) in TAD boundaries and A/B compartmentalization as compared to controls. However, Fit Hi-C, a method to analyze genome-wide chromosomal contacts that are statistically significant, revealed that interactions and accessibility at a large number of enhancer regions are changed in *CTCF-CKO* mice: 4,037 increase/decrease in contact compared to 1,013 unchanged. The genes in the surrounding chromosomal regions are enriched for cardiac pathology pathways (Rosa-Garrido et al., 2017).

Taken together, these experiments suggest that the preexisting chromatin landscape, which has existed before the loss of TADs, is sufficient to retain the DNA-TF accessibility even when the TADs are lost. TADs and their associated CTCF sites alone might not be essential for correct developmental gene expression; however, they can cause misexpression when redirected with different loci being more or less sensitive to these changes, leading to embryonic lethality in the mouse models tested.

An important facilitator of TAD formation is the four-subunit protein complex, cohesin, well studied for its role in chromatid formation and chromosome segregation during mitosis (Lica et al., 1986; Cooke et al., 1987). The cohesin complex forms a ring-like structure and consists of core proteins: structural maintenance of chromosomes (SMC)1, SMC3, RAD21, and stromal antigen (STAG)1/2. Auxin-induced degradation of the cohesin subunit RAD21 in human cancer cell lines leads to a loss of all TADs, but transcriptional activity remains largely unchanged (Rao et al., 2017). Another subunit of the cohesin core complex, STAG2, is required for heart morphogenesis; *Stag2*-null embryos die by E10.5 and exhibit observable heart abnormalities by E9.5 (De Koninck et al., 2020). Histological analysis of *Stag2* knockout embryos at E9.5 shows significantly smaller right ventricles and shorter outflow tracts, with decreased anti-phosphohistone H3 staining associated with reduced CM proliferation when compared to controls (De Koninck et al., 2020). Based on observed right ventricle and outflow tract defects, authors examined SHF progenitor populations in *Stag2*-knockout embryos, and RNA-seq analysis indicated downregulation of important SHF regulators, *Fgf8*, *Hand2*, and *Wnt5a* (De Koninck et al., 2020). Further work is required to understand the molecular function of the cohesin complex of proteins, their contribution to TAD formation, and their role in cardiac disease.

### Non-coding RNAs

Long non-coding RNAs (lncRNAs) are greater than 200 nucleotides (nt) in length and function by binding DNA, other RNAs, or RNA binding proteins or can contain their own transcriptional start site to make micropeptides (<100 amino acids) (Ulitsky and Bartel, 2013). There are also reports of lncRNAs interacting with established chromatin modifiers (Mishra and Kanduri, 2019). The first instance of this is HOTAIR (*HOX transcript antisense intergenic RNA*), which is transcribed from the human *HOXC* locus to *trans* regulate transcriptional silencing of the *HOXD* locus in a tissue-dependent manner by direct interaction with the histone methyltransferase PRC2 (Rinn et al., 2007). Further experiments reveal that 20% of human lncRNAs are associated with PRC2 (Khalil et al., 2009), including the well-characterized lncRNAs *Xist*, *RepA*, *Kcnq1ot1*, *Braveheart*, and *Malat-1*, suggesting that lncRNAs serve to modulate a scanning of the genome for genes that require silencing (Davidovich and Cech, 2015), which could be facilitated by PRC2 binding nascent RNA (Beltran et al., 2016). The biological relevance of lncRNA-mediated chromatin modulation has also been demonstrated by mis-expression of lncRNAs in human cancer (Begolli et al., 2019).

Perhaps more interesting are a number of studies that indicate that lncRNAs directly interact with the zinc finger TF CTCF and that these RNA–CTCF interactions are required for some TADs to form (Saldana-Meyer et al., 2014; Kung et al., 2015; Hansen et al., 2019). Work from the Reinberg lab generated a CTCF mutant protein that retains the DNA binding capability but has decreased RNA binding by deleting 14 amino acids from zinc finger 1 and nine amino acids from zinc finger 10 (Saldana-Meyer et al., 2019). Using mESCs with an AID system (Nora et al., 2017) to knock out CTCF and express the RNA binding-deficient CTCF mutant protein, single-cell RNA-seq experiments along with Hi-C analysis identified only modest changes in both gene expression and 3D chromatin structure within CTCF RNA binding-deficient cell lines, with 60% of TF sites exhibiting decreased CTCF binding within gene promoters and no changes in A/B compartmentalization (Saldana-Meyer et al., 2019). The Tjian lab addressed the same question by deleting the internal RNA binding region (RBR_*i*_) of CTCF protein in endogenous mESC lines (Hansen et al., 2019). RNA-seq analysis indicates that RBR_*i*_ deleted cell lines show modest gene expression changes (∼500 genes mis-regulated with an average fold change of 2.7) and no change in A/B compartmentalization similar to what was seen by Saldana-Meyer et al. Chromatin conformation capture experiments find that almost half of all CTCF loops are lost in RBR_*i*_ deletion mutants, suggesting that there are two kinds of CTCF-mediated loops: RBR_*i*_ dependent and RBR_*i*_ independent. Even with modest gene expression changes in RBR_*i*_ deletion mutants, some TAD formation requires RNA binding with CTCF (Hansen et al., 2019). Although these direct RNA–CTCF interactions are still controversial in the field, it is possible that these interactions might explain how lncRNAs help modulate gene expression.

In the context of cardiac development, a specific role of lncRNAs has been uncovered (Martens et al., 2017; Hobuss et al., 2019). The *Myh*-associated RNA transcripts, or *Myheart* (*Mhrt*), are alternatively spliced lncRNA transcripts that lie within the *myosin* gene locus (Han et al., 2014). This well-characterized lncRNA is downregulated in hearts pressure-overloaded by transaortic constriction (TAC). Overexpression of *Mhrt* led to cardioprotective effects with minimal/absent fibrosis, improvement in fractional shortening, normalized left ventricle size, and reduced change in *Nppa* expression. Luciferase assays and ChIP experiments show that the *Mhrt* promoter is directly regulated by the chromatin remodeling factor *Brg1*, the ATPase subunit of SWI2/SNF2-like chromatin-remodeling complexes. RNA immunoprecipitation experiments demonstrate that *Mhrt* directly binds to BRG1 to reduce its occupancy at target genes (Han et al., 2014).

Recent work from the Bruneau lab employed RNA-seq reads of mESCs differentiated into CMs to screen for lncRNAs on the basis of their epigenetic regulation, clear splice structure, homology to human and/or mammalian genomes, cardiac progenitor specificity, and expression in the developing embryo (George et al., 2019). Six novel lncRNAs were identified: *Rubie*, *Handlr*, *Atcayos*, *HrtLincR4*, *HrtLincR5*, and *HrtLincRX*. Knockout mouse lines were generated using CRISPR/Cas9 genome editing and assayed for loss of expression or cardiac phenotypes. Authors report that none of the tested lncRNAs are required for viable mouse development, suggesting a lack of function or that, within the context of cardiac development, further understanding of lncRNA biology would require manipulation of additional molecular compensatory mechanisms (George et al., 2019).

Short (<22-nt) single-stranded non-coding RNA molecules known as microRNAs (miRNAs) have also been implicated in epigenomic regulation (Yao et al., 2019). The role that miRNAs play in cardiovascular diseases has been extensively covered by recent reviews (Colpaert and Calore, 2021).

## Cardiac Diseases and the Epigenome

Epigenetic changes have been identified as a causative agent for disease in multiple organ systems and cell types (Zoghbi and Beaudet, 2016). Germline mutations in genes encoding part of the cohesin complex and its regulatory factors are collectively referred to as cohesinopathies (Piche et al., 2019). The most common of these is the Cornelia de Lange syndrome (CdLS, OMIM 122470) where patients present with growth retardation, intellectual disability, and facial dysmorphism (Sarogni et al., 2020). Of the CdLS patients, 14–70% also present with congenital heart defects (Chatfield et al., 2012). Sixty percent of patients carry heterozygous mutations in NIPBL, a protein that loads cohesin onto chromatin (Liu et al., 2009). Inducible pluripotent stem cells (iPSCs) derived from CdLS patients were differentiated to CMs, and RNA-seq analysis identified altered gene expression in several critical cardiac development genes: *GATA4/6*, *MYH6/7*, *MYH7*, *ACTN2*, *HAND2*, *TBX1/5*, and *TDGF1*, within the *NIPBL* haploinsufficient samples as compared to control patient samples (Mills et al., 2018).

The DiGeorge syndrome, a 1.5- to 3.0-Mb heterozygous deletion of chromosome 22q11 (OMIM 188400), causes congenital heart defects and is linked to *TBX1* haploinsufficiency (Lindsay et al., 1999). *Tbx1* is expressed in the SHF, and patients with this disease show outflow tract defects and persistent truncus arteriosus (lack of septation between the aorta and pulmonary trunk) (Du et al., 2019). TBX1 regulates chromatin by interacting with various epigenetic modifiers: the BAF60A/SMARCD1 subunits, the Setd7 histone H3K4 monomethyltransferase, and the histone demethylase LSD1 (Chen et al., 2012; Fulcoli et al., 2016). The importance of epigenetic regulation in this disease is demonstrated by treating pregnant mice with a histone demethylase inhibitor, thus increasing levels of methylated H3K4, which partially rescues the cardiovascular anomalies in *Tbx1*^+/KO^ embryos (Fulcoli et al., 2016).

Children with Down syndrome (trisomy 21; OMIM 190685) present with a higher-than-normal incidence rate (>50%) of ventricular septal and atrial septal defects (Antonarakis, 2017). Transcriptomic analysis of 45 trisomy 21 patient samples identified significant misexpression of 247 genes not located on chromosome 21, compared to only 77 genes dysregulated that are located on chromosome 21 (Vilardell et al., 2011). Using patient samples from a pair of monozygotic twins, one of which had trisomy 21, Letourneau et al. (2014) performed transcriptomic analysis and derived iPSC cell lines. Domains of dysregulated genes are identified throughout the genome that overlaps with nuclear lamina associating regions of low gene expression, suggesting that chromatin modulation might be responsible for gene mis-regulation in this syndrome (Letourneau et al., 2014). However, it is still unclear how cardiac defects arise from these global chromatin landscape changes.

CHD7, a chromatin remodeling factor that is a member of the chromodomain helicase DNA-binding family of ATP-dependent chromatin remodeling enzymes, is mutated in the CHARGE syndrome (coloboma of the eye, heart defects, atresia of the choanae, severe retardation of growth/development, genital abnormalities, and ear abnormalities; OMIM 214800) (Vissers et al., 2004). Nonsense and frameshift indel mutations in CHD7 occur *de novo*, resulting in the generation of a loss-of-function protein (Basson and van Ravenswaaij-Arts, 2015). Seventy-five percent of patients present with a congenital heart defect (Lalani et al., 2006). ChIP-qPCR analysis on NkL-Tag, a mouse cardiac cell line, indicates that CHD7 binds directly to *Nkx2-5* enhancers *in vitro* (Liu et al., 2014). Further ChIP analysis demonstrates that recruitment to *Nkx2-5* enhancers is mediated via the CHD7 interaction with SMAD1 downstream of BMP2 signaling (Liu et al., 2014).

Other than congenital cardiac disease, postnatal epigenetic changes are also correlated with gene expression changes leading to cardiovascular aging, a complex process characterized by decreased heart function and ventricular and atrial remodeling (Zhang et al., 2018). These epigenetic changes are thought to be induced by changes in reactive oxygen species (ROS) or metabolite levels (Etchegaray and Mostoslavsky, 2016). Myocardial infarction or pressure overload conditions in the heart result in fibroblast activation and inflammation, leading to cardiac fibrosis, which causes changes in epigenetic regulation (Felisbino and McKinsey, 2018). We have reviewed these changes in earlier relevant sections, but the etiology of how these epigenetic changes lead to cardiac dysfunction requires further study.

## Conclusion

Extensive work in recent years has uncovered some of the molecular mechanisms which control chromatin regulation and disease conditions arising from epigenetic mis-regulation. The conserved nature of chromatin domains and the regulatory mechanisms controlling their establishment and maintenance would suggest that changes to chromatin landscape would lead to dramatic changes in gene expression. However, for the most part, this has not been the case, suggesting that in an epigenetic context, overlapping layers of modifications regulate transcription. Although our understanding of chromatin biology has vastly advanced in recent years with chromatin conformation capture technologies, much remains unclear about what the functional role is of these domains in regulating enhancer accessibility or enabling/repressing transcription. In adult disease conditions, epigenetic changes play a more complex role with a large variability of phenotypes between patients, further confounding the analysis of causative factors. Alterations to epigenetic factors that correlate with disease suggest that these aberrant proteins undergo changes in subunit function and lack biological redundancies. Interestingly, cardiomyopathy phenotypes in human patients appear to be sensitive to these epigenetic aberrations, suggesting specificity in both tissue and type of epigenetic factor expressed during heart development. Refining our understanding of the epigenetic mechanisms at play in cardiac development is required to parse out these phenotypes.

## Author Contributions

Both authors conceptualized, wrote, and edited the manuscript.

## Conflict of Interest

The authors declare that the research was conducted in the absence of any commercial or financial relationships that could be construed as a potential conflict of interest.

## References

[B1] Akerberg B. N. Gu F. VanDusen N. J. Zhang X. Dong R. Li K. (2019). A reference map of murine cardiac transcription factor chromatin occupancy identifies dynamic and conserved enhancers. *Nat. Commun.* 10:4907. 10.1038/s41467-019-12812-3 31659164 PMC6817842

[B2] Alipour E. Marko J. F. (2012). Self-organization of domain structures by DNA-loop-extruding enzymes. *Nucleic Acids Res.* 40 11202–11212. 10.1093/nar/gks925 23074191 PMC3526278

[B3] Allfrey V. G. Faulkner R. Mirsky A. E. (1964). Acetylation and Methylation of Histones and Their Possible Role in the Regulation of Rna Synthesis. *Proc. Natl. Acad. Sci. U S A.* 51 786–794. 10.1073/pnas.51.5.786 14172992 PMC300163

[B4] Antonarakis S. E. (2017). Down syndrome and the complexity of genome dosage imbalance. *Nat. Rev. Genet.* 18 147–163. 10.1038/nrg.2016.154 28029161

[B5] Barski A. Cuddapah S. Cui K. Roh T. Y. Schones D. E. Wang Z. (2007). High-resolution profiling of histone methylations in the human genome. *Cell* 129 823–837. 10.1016/j.cell.2007.05.009 17512414

[B6] Basson M. A. van Ravenswaaij-Arts C. (2015). Functional Insights into Chromatin Remodelling from Studies on CHARGE Syndrome. *Trends Genet.* 31 600–611. 10.1016/j.tig.2015.05.009 26411921 PMC4604214

[B7] Begolli R. Sideris N. Giakountis A. (2019). LncRNAs as Chromatin Regulators in Cancer: From Molecular Function to Clinical Potential. *Cancers* 11:11101524. 10.3390/cancers11101524 31658672 PMC6826483

[B8] Beltran M. Yates C. M. Skalska L. Dawson M. Reis F. P. Viiri K. (2016). The interaction of PRC2 with RNA or chromatin is mutually antagonistic. *Genome Res.* 26 896–907. 10.1101/gr.197632.115 27197219 PMC4937559

[B9] Bird A. P. (1978). Use of restriction enzymes to study eukaryotic DNA methylation: II. The symmetry of methylated sites supports semi-conservative copying of the methylation pattern. *J. Mol. Biol.* 118 49–60. 10.1016/0022-2836(78)90243-7 625057

[B10] Black B. L. (2007). Transcriptional pathways in second heart field development. *Semin. Cell Dev. Biol.* 18 67–76. 10.1016/j.semcdb.2007.01.001 17276708 PMC1855211

[B11] Bruneau B. G. (2013). Signaling and transcriptional networks in heart development and regeneration. *Cold Spring Harb. Perspect. Biol.* 5:a008292. 10.1101/cshperspect.a008292 23457256 PMC3578359

[B12] Chamberlain A. A. Lin M. Lister R. L. Maslov A. A. Wang Y. Suzuki M. (2014). DNA methylation is developmentally regulated for genes essential for cardiogenesis. *J. Am. Heart. Assoc.* 3:e000976. 10.1161/JAHA.114.000976 24947998 PMC4309105

[B13] Chatfield K. C. Schrier S. A. Li J. Clark D. Kaur M. Kline A. D. (2012). Congenital heart disease in Cornelia de Lange syndrome: phenotype and genotype analysis. *Am. J. Med. Genet. A* 158A 2499–2505. 10.1002/ajmg.a.35582 22965847 PMC3551981

[B14] Chen L. Fulcoli F. G. Ferrentino R. Martucciello S. Illingworth E. A. Baldini A. (2012). Transcriptional control in cardiac progenitors: Tbx1 interacts with the BAF chromatin remodeling complex and regulates Wnt5a. *PLoS Genet.* 8:e1002571. 10.1371/journal.pgen.1002571 22438823 PMC3305383

[B15] Christoffels V. Jensen B. (2020). Cardiac Morphogenesis: Specification of the Four-Chambered Heart. *Cold Spring Harb. Perspect. Biol.* 12:37143. 10.1101/cshperspect.a037143 31932321 PMC7528854

[B16] Clapier C. R. Cairns B. R. (2009). The biology of chromatin remodeling complexes. *Annu. Rev. Biochem.* 78 273–304. 10.1146/annurev.biochem.77.062706.153223 19355820

[B17] Colpaert R. M. W. Calore M. (2021). Epigenetics and microRNAs in cardiovascular diseases. *Genomics* 113 540–551. 10.1016/j.ygeno.2020.12.042 33482325

[B18] Cooke C. A. Heck M. M. Earnshaw W. C. (1987). The inner centromere protein (INCENP) antigens: movement from inner centromere to midbody during mitosis. *J. Cell Biol.* 105 2053–2067. 10.1083/jcb.105.5.2053 3316246 PMC2114862

[B19] Dali R. Blanchette M. (2017). A critical assessment of topologically associating domain prediction tools. *Nucleic Acids Res.* 45 2994–3005. 10.1093/nar/gkx145 28334773 PMC5389712

[B20] Davidovich C. Cech T. R. (2015). The recruitment of chromatin modifiers by long noncoding RNAs: lessons from PRC2. *RNA* 21 2007–2022. 10.1261/rna.053918.115 26574518 PMC4647455

[B21] De Koninck M. Lapi E. Badia-Careaga C. Cossio I. Gimenez-Llorente D. Rodriguez-Corsino M. (2020). Essential Roles of Cohesin STAG2 in Mouse Embryonic Development and Adult Tissue Homeostasis. *Cell Rep.* 32:108014. 10.1016/j.celrep.2020.108014 32783938

[B22] Dixon J. R. Selvaraj S. Yue F. Kim A. Li Y. Shen Y. (2012). Topological domains in mammalian genomes identified by analysis of chromatin interactions. *Nature* 485 376–380. 10.1038/nature11082 22495300 PMC3356448

[B23] Du Q. de la Morena M. T. van Oers N. S. C. (2019). The Genetics and Epigenetics of 22q11.2 Deletion Syndrome. *Front. Genet.* 10:1365. 10.3389/fgene.2019.01365 32117416 PMC7016268

[B24] Eres I. E. Gilad Y. (2020). A TAD Skeptic: Is 3D Genome Topology Conserved? *Trends Genet*. 37 216–223. 10.1016/j.tig.2020.10.009 33203573 PMC8120795

[B25] Ernst J. Kheradpour P. Mikkelsen T. S. Shoresh N. Ward L. D. Epstein C. B. (2011). Mapping and analysis of chromatin state dynamics in nine human cell types. *Nature* 473 43–49. 10.1038/nature09906 21441907 PMC3088773

[B26] Etchegaray J. P. Mostoslavsky R. (2016). Interplay between Metabolism and Epigenetics: A Nuclear Adaptation to Environmental Changes. *Mol. Cell* 62 695–711. 10.1016/j.molcel.2016.05.029 27259202 PMC4893201

[B27] Fang X. Poulsen R. R. Wang-Hu J. Shi O. Calvo N. S. Simmons C. S. (2016). Knockdown of DNA methyltransferase 3a alters gene expression and inhibits function of embryonic cardiomyocytes. *FASEB J.* 30 3238–3255. 10.1096/fj.201600346R 27306334 PMC5001511

[B28] Felisbino M. B. McKinsey T. A. (2018). Epigenetics in Cardiac Fibrosis: Emphasis on Inflammation and Fibroblast Activation. *JACC Basic Transl. Sci.* 3 704–715. 10.1016/j.jacbts.2018.05.003 30456341 PMC6234501

[B29] Felsenfeld G. Groudine M. (2003). Controlling the double helix. *Nature* 421 448–453. 10.1038/nature01411 12540921

[B30] Fudenberg G. Imakaev M. Lu C. Goloborodko A. Abdennur N. Mirny L. A. (2016). Formation of Chromosomal Domains by Loop Extrusion. *Cell Rep.* 15 2038–2049. 10.1016/j.celrep.2016.04.085 27210764 PMC4889513

[B31] Fulcoli F. G. Franzese M. Liu X. Zhang Z. Angelini C. Baldini A. (2016). Rebalancing gene haploinsufficiency in vivo by targeting chromatin. *Nat. Commun.* 7:11688. 10.1038/ncomms11688 27256596 PMC4895808

[B32] George M. R. Duan Q. Nagle A. Kathiriya I. S. Huang Y. Rao K. (2019). Minimal in vivo requirements for developmentally regulated cardiac long intergenic non-coding RNAs. *Development* 146 185314. 10.1242/dev.185314 31784461 PMC6918742

[B33] Gilsbach R. Preissl S. Gruning B. A. Schnick T. Burger L. Benes V. (2014). Dynamic DNA methylation orchestrates cardiomyocyte development, maturation and disease. *Nat. Commun.* 5:5288. 10.1038/ncomms6288 25335909 PMC4220495

[B34] Gilsbach R. Schwaderer M. Preissl S. Gruning B. A. Kranzhofer D. Schneider P. (2018). Distinct epigenetic programs regulate cardiac myocyte development and disease in the human heart in vivo. *Nat. Commun.* 9:391. 10.1038/s41467-017-02762-z 29374152 PMC5786002

[B35] Gomez-Velazquez M. Badia-Careaga C. Lechuga-Vieco A. V. Nieto-Arellano R. Tena J. J. Rollan I. (2017). CTCF counter-regulates cardiomyocyte development and maturation programs in the embryonic heart. *PLoS Genet.* 13:e1006985. 10.1371/journal.pgen.1006985 28846746 PMC5591014

[B36] Greenberg M. V. C. Bourc’his D. (2019). The diverse roles of DNA methylation in mammalian development and disease. *Nat. Rev. Mol. Cell Biol.* 20 590–607. 10.1038/s41580-019-0159-6 31399642

[B37] Grubert F. Srivas R. Spacek D. V. Kasowski M. Ruiz-Velasco M. Sinnott-Armstrong N. (2020). Landscape of cohesin-mediated chromatin loops in the human genome. *Nature* 583 737–743. 10.1038/s41586-020-2151-x 32728247 PMC7410831

[B38] Haas J. Frese K. S. Park Y. J. Keller A. Vogel B. Lindroth A. M. (2013). Alterations in cardiac DNA methylation in human dilated cardiomyopathy. *EMBO Mol. Med.* 5 413–429. 10.1002/emmm.201201553 23341106 PMC3598081

[B39] Han P. Li W. Lin C. H. Yang J. Shang C. Nuernberg S. T. (2014). A long noncoding RNA protects the heart from pathological hypertrophy. *Nature* 514 102–106. 10.1038/nature13596 25119045 PMC4184960

[B40] Hang C. T. Yang J. Han P. Cheng H. L. Shang C. Ashley E. (2010). Chromatin regulation by Brg1 underlies heart muscle development and disease. *Nature* 466 62–67. 10.1038/nature09130 20596014 PMC2898892

[B41] Hansen A. S. Hsieh T. S. Cattoglio C. Pustova I. Saldana-Meyer R. Reinberg D. (2019). Distinct Classes of Chromatin Loops Revealed by Deletion of an RNA-Binding Region in CTCF. *Mol. Cell* 76:39. e313. 10.1016/j.molcel.2019.07.039 31522987 PMC7251926

[B42] Hansen A. S. Pustova I. Cattoglio C. Tjian R. Darzacq X. (2017). CTCF and cohesin regulate chromatin loop stability with distinct dynamics. *Elife* 6:25776. 10.7554/eLife.25776 28467304 PMC5446243

[B43] Hargreaves D. C. Crabtree G. R. (2011). ATP-dependent chromatin remodeling: genetics, genomics and mechanisms. *Cell Res.* 21 396–420. 10.1038/cr.2011.32 21358755 PMC3110148

[B44] Harmston N. Ing-Simmons E. Tan G. Perry M. Merkenschlager M. Lenhard B. (2017). Topologically associating domains are ancient features that coincide with Metazoan clusters of extreme noncoding conservation. *Nat. Commun.* 8:441. 10.1038/s41467-017-00524-5 28874668 PMC5585340

[B45] He A. Ma Q. Cao J. von Gise A. Zhou P. Xie H. (2012). Polycomb repressive complex 2 regulates normal development of the mouse heart. *Circ. Res.* 110 406–415. 10.1161/CIRCRESAHA.111.252205 22158708 PMC3282145

[B46] He L. Tian X. Zhang H. Hu T. Huang X. Zhang L. (2014). BAF200 is required for heart morphogenesis and coronary artery development. *PLoS One* 9:e109493. 10.1371/journal.pone.0109493 25299188 PMC4192121

[B47] Ho L. Crabtree G. R. (2010). Chromatin remodelling during development. *Nature* 463 474–484. 10.1038/nature08911 20110991 PMC3060774

[B48] Hobuss L. Bar C. Thum T. (2019). Long Non-coding RNAs: At the Heart of Cardiac Dysfunction? *Front. Physiol.* 10:30. 10.3389/fphys.2019.00030 30761015 PMC6361744

[B49] Hoff K. Lemme M. Kahlert A. K. Runde K. Audain E. Schuster D. (2019). DNA methylation profiling allows for characterization of atrial and ventricular cardiac tissues and hiPSC-CMs. *Clin. Epigenet.* 11:89. 10.1186/s13148-019-0679-0 31186048 PMC6560887

[B50] Ibn-Salem J. Kohler S. Love M. I. Chung H. R. Huang N. Hurles M. E. (2014). Deletions of chromosomal regulatory boundaries are associated with congenital disease. *Genome Biol.* 15:423. 10.1186/s13059-014-0423-1 25315429 PMC4180961

[B51] Karlic R. Chung H. R. Lasserre J. Vlahovicek K. Vingron M. (2010). Histone modification levels are predictive for gene expression. *Proc. Natl. Acad. Sci. U S A.* 107 2926–2931. 10.1073/pnas.0909344107 20133639 PMC2814872

[B52] Kelly R. G. Buckingham M. E. Moorman A. F. (2014). Heart fields and cardiac morphogenesis. *Cold Spring Harb. Perspect. Med.* 4:a015750. 10.1101/cshperspect.a015750 25274757 PMC4200205

[B53] Kempfer R. Pombo A. (2020). Methods for mapping 3D chromosome architecture. *Nat. Rev. Genet.* 21 207–226. 10.1038/s41576-019-0195-2 31848476

[B54] Khalil A. M. Guttman M. Huarte M. Garber M. Raj A. Rivea Morales D. (2009). Many human large intergenic noncoding RNAs associate with chromatin-modifying complexes and affect gene expression. *Proc. Natl. Acad. Sci. U S A.* 106 11667–11672. 10.1073/pnas.0904715106 19571010 PMC2704857

[B55] Kornberg R. D. (1974). Chromatin structure: a repeating unit of histones and DNA. *Science* 184 868–871. 10.1126/science.184.4139.868 4825889

[B56] Krefting J. Andrade-Navarro M. A. Ibn-Salem J. (2018). Evolutionary stability of topologically associating domains is associated with conserved gene regulation. *BMC Biol.* 16:87. 10.1186/s12915-018-0556-x 30086749 PMC6091198

[B57] Kung J. T. Kesner B. An J. Y. Ahn J. Y. Cifuentes-Rojas C. Colognori D. (2015). Locus-specific targeting to the X chromosome revealed by the RNA interactome of CTCF. *Mol. Cell* 57 361–375. 10.1016/j.molcel.2014.12.006 25578877 PMC4316200

[B58] Lalani S. R. Safiullah A. M. Fernbach S. D. Harutyunyan K. G. Thaller C. Peterson L. E. (2006). Spectrum of CHD7 mutations in 110 individuals with CHARGE syndrome and genotype-phenotype correlation. *Am. J. Hum. Genet.* 78 303–314. 10.1086/500273 16400610 PMC1380237

[B59] Letourneau A. Santoni F. A. Bonilla X. Sailani M. R. Gonzalez D. Kind J. (2014). Domains of genome-wide gene expression dysregulation in Down’s syndrome. *Nature* 508 345–350. 10.1038/nature13200 24740065

[B60] Li X. Yue X. Pastor W. A. Lin L. Georges R. Chavez L. (2016). Tet proteins influence the balance between neuroectodermal and mesodermal fate choice by inhibiting Wnt signaling. *Proc. Natl. Acad. Sci. U S A.* 113 E8267–E8276. 10.1073/pnas.1617802113 27930333 PMC5187696

[B61] Lica L. M. Narayanswami S. Hamkalo B. A. (1986). Mouse satellite DNA, centromere structure, and sister chromatid pairing. *J. Cell Biol.* 103 1145–1151. 10.1083/jcb.103.4.1145 2429969 PMC2114340

[B62] Lickert H. Takeuchi J. K. Von Both I. Walls J. R. McAuliffe F. Adamson S. L. (2004). Baf60c is essential for function of BAF chromatin remodelling complexes in heart development. *Nature* 432 107–112. 10.1038/nature03071 15525990

[B63] Lieberman-Aiden E. van Berkum N. L. Williams L. Imakaev M. Ragoczy T. Telling A. (2009). Comprehensive mapping of long-range interactions reveals folding principles of the human genome. *Science* 326 289–293. 10.1126/science.1181369 19815776 PMC2858594

[B64] Lindsay E. A. Botta A. Jurecic V. Carattini-Rivera S. Cheah Y. C. Rosenblatt H. M. (1999). Congenital heart disease in mice deficient for the DiGeorge syndrome region. *Nature* 401 379–383. 10.1038/43900 10517636

[B65] Liu J. Zhang Z. Bando M. Itoh T. Deardorff M. A. Clark D. (2009). Transcriptional dysregulation in NIPBL and cohesin mutant human cells. *PLoS Biol.* 7:e1000119. 10.1371/journal.pbio.1000119 19468298 PMC2680332

[B66] Liu Y. Harmelink C. Peng Y. Chen Y. Wang Q. Jiao K. (2014). CHD7 interacts with BMP R-SMADs to epigenetically regulate cardiogenesis in mice. *Hum. Mol. Genet.* 23 2145–2156. 10.1093/hmg/ddt610 24293546 PMC3959819

[B67] Lupianez D. G. Kraft K. Heinrich V. Krawitz P. Brancati F. Klopocki E. (2015). Disruptions of topological chromatin domains cause pathogenic rewiring of gene-enhancer interactions. *Cell* 161 1012–1025. 10.1016/j.cell.2015.04.004 25959774 PMC4791538

[B68] Lupianez D. G. Spielmann M. Mundlos S. (2016). Breaking TADs: How Alterations of Chromatin Domains Result in Disease. *Trends Genet.* 32 225–237. 10.1016/j.tig.2016.01.003 26862051

[B69] Martens L. Ruhle F. Stoll M. (2017). LncRNA secondary structure in the cardiovascular system. *Noncoding RNA Res.* 2 137–142. 10.1016/j.ncrna.2017.12.001 30159432 PMC6084829

[B70] McGhee J. D. Felsenfeld G. (1980). Nucleosome structure. *Annu. Rev. Biochem.* 49 1115–1156. 10.1146/annurev.bi.49.070180.005343 6996562

[B71] Meder B. Haas J. Sedaghat-Hamedani F. Kayvanpour E. Frese K. Lai A. (2017). Epigenome-Wide Association Study Identifies Cardiac Gene Patterning and a Novel Class of Biomarkers for Heart Failure. *Circulation* 136 1528–1544. 10.1161/CIRCULATIONAHA.117.027355 28838933

[B72] Mills J. A. Herrera P. S. Kaur M. Leo L. McEldrew D. Tintos-Hernandez J. A. (2018). NIPBL(+/-) haploinsufficiency reveals a constellation of transcriptome disruptions in the pluripotent and cardiac states. *Sci. Rep.* 8:1056. 10.1038/s41598-018-19173-9 29348408 PMC5773608

[B73] Mishra K. Kanduri C. (2019). Understanding Long Noncoding RNA and Chromatin Interactions: What We Know So Far. *Noncoding RNA* 5:5040054. 10.3390/ncrna5040054 31817041 PMC6958424

[B74] Morawska M. Ulrich H. D. (2013). An expanded tool kit for the auxin-inducible degron system in budding yeast. *Yeast* 30 341–351. 10.1002/yea.2967 23836714 PMC4171812

[B75] Movassagh M. Vujic A. Foo R. (2011). Genome-wide DNA methylation in human heart failure. *Epigenomics* 3 103–109. 10.2217/epi.10.70 22126157

[B76] Muro E. M. Ibn-Salem J. Andrade-Navarro M. A. (2019). The distributions of protein coding genes within chromatin domains in relation to human disease. *Epigenet. Chromatin* 12:72. 10.1186/s13072-019-0317-2 31805995 PMC6894242

[B77] Nora E. P. Caccianini L. Fudenberg G. So K. Kameswaran V. Nagle A. (2020). Molecular basis of CTCF binding polarity in genome folding. *Nat. Commun.* 11:5612. 10.1038/s41467-020-19283-x 33154377 PMC7645679

[B78] Nora E. P. Goloborodko A. Valton A. L. Gibcus J. H. Uebersohn A. Abdennur N. (2017). Targeted Degradation of CTCF Decouples Local Insulation of Chromosome Domains from Genomic Compartmentalization. *Cell* 169 930–944e922. 10.1016/j.cell.2017.05.004 28525758 PMC5538188

[B79] Nora E. P. Lajoie B. R. Schulz E. G. Giorgetti L. Okamoto I. Servant N. (2012). Spatial partitioning of the regulatory landscape of the X-inactivation centre. *Nature* 485 381–385. 10.1038/nature11049 22495304 PMC3555144

[B80] Nord A. S. Blow M. J. Attanasio C. Akiyama J. A. Holt A. Hosseini R. (2013). Rapid and pervasive changes in genome-wide enhancer usage during mammalian development. *Cell* 155 1521–1531. 10.1016/j.cell.2013.11.033 24360275 PMC3989111

[B81] Olson E. N. (2006). Gene regulatory networks in the evolution and development of the heart. *Science* 313 1922–1927. 10.1126/science.1132292 17008524 PMC4459601

[B82] Papait R. Cattaneo P. Kunderfranco P. Greco C. Carullo P. Guffanti A. (2013). Genome-wide analysis of histone marks identifying an epigenetic signature of promoters and enhancers underlying cardiac hypertrophy. *Proc. Natl. Acad. Sci. U S A.* 110 20164–20169. 10.1073/pnas.1315155110 24284169 PMC3864351

[B83] Pepin M. E. Ha C. M. Crossman D. K. Litovsky S. H. Varambally S. Barchue J. P. (2019). Genome-wide DNA methylation encodes cardiac transcriptional reprogramming in human ischemic heart failure. *Lab. Invest.* 99 371–386. 10.1038/s41374-018-0104-x 30089854 PMC6515060

[B84] Piche J. Van Vliet P. P. Puceat M. Andelfinger G. (2019). The expanding phenotypes of cohesinopathies: one ring to rule them all! *Cell Cycle* 18 2828–2848. 10.1080/15384101.2019.1658476 31516082 PMC6791706

[B85] Pinto A. R. Ilinykh A. Ivey M. J. Kuwabara J. T. D’Antoni M. L. Debuque R. (2016). Revisiting Cardiac Cellular Composition. *Circ. Res.* 118 400–409. 10.1161/CIRCRESAHA.115.307778 26635390 PMC4744092

[B86] Rada-Iglesias A. Bajpai R. Swigut T. Brugmann S. A. Flynn R. A. Wysocka J. (2011). A unique chromatin signature uncovers early developmental enhancers in humans. *Nature* 470 279–283. 10.1038/nature09692 21160473 PMC4445674

[B87] Rao S. S. P. Huang S. C. Glenn, St Hilaire B. Engreitz J. M. Perez E. M. (2017). Cohesin Loss Eliminates All Loop Domains. *Cell* 171 305–320e324. 10.1016/j.cell.2017.09.026 28985562 PMC5846482

[B88] Rao S. S. Huntley M. H. Durand N. C. Stamenova E. K. Bochkov I. D. Robinson J. T. (2014). A 3D map of the human genome at kilobase resolution reveals principles of chromatin looping. *Cell* 159 1665–1680. 10.1016/j.cell.2014.11.021 25497547 PMC5635824

[B89] Riggs A. D. (1975). X inactivation, differentiation, and DNA methylation. *Cytogenet. Cell Genet.* 14 9–25. 10.1159/000130315 1093816

[B90] Rinn J. L. Kertesz M. Wang J. K. Squazzo S. L. Xu X. Brugmann S. A. (2007). Functional demarcation of active and silent chromatin domains in human HOX loci by noncoding RNAs. *Cell* 129 1311–1323. 10.1016/j.cell.2007.05.022 17604720 PMC2084369

[B91] Rosa-Garrido M. Chapski D. J. Schmitt A. D. Kimball T. H. Karbassi E. Monte E. (2017). High-Resolution Mapping of Chromatin Conformation in Cardiac Myocytes Reveals Structural Remodeling of the Epigenome in Heart Failure. *Circulation* 136 1613–1625. 10.1161/CIRCULATIONAHA.117.029430 28802249 PMC5648689

[B92] Saldana-Meyer R. Gonzalez-Buendia E. Guerrero G. Narendra V. Bonasio R. Recillas-Targa F. (2014). CTCF regulates the human p53 gene through direct interaction with its natural antisense transcript, Wrap53. *Genes Dev.* 28 723–734. 10.1101/gad.236869.113 24696455 PMC4015496

[B93] Saldana-Meyer R. Rodriguez-Hernaez J. Escobar T. Nishana M. Jacome-Lopez K. Nora E. P. (2019). RNA Interactions Are Essential for CTCF-Mediated Genome Organization. *Mol. Cell* 76 412–422e415. 10.1016/j.molcel.2019.08.015 31522988 PMC7195841

[B94] Sanborn A. L. Rao S. S. Huang S. C. Durand N. C. Huntley M. H. Jewett A. I. (2015). Chromatin extrusion explains key features of loop and domain formation in wild-type and engineered genomes. *Proc. Natl. Acad. Sci. U S A.* 112 E6456–E6465. 10.1073/pnas.1518552112 26499245 PMC4664323

[B95] Sarogni P. Pallotta M. M. Musio A. (2020). Cornelia de Lange syndrome: from molecular diagnosis to therapeutic approach. *J. Med. Genet.* 57 289–295. 10.1136/jmedgenet-2019-106277 31704779 PMC7231464

[B96] Shikama N. Lutz W. Kretzschmar R. Sauter N. Roth J. F. Marino S. (2003). Essential function of p300 acetyltransferase activity in heart, lung and small intestine formation. *EMBO J.* 22 5175–5185. 10.1093/emboj/cdg502 14517255 PMC204485

[B97] Stankunas K. Hang C. T. Tsun Z. Y. Chen H. Lee N. V. Wu J. I. (2008). Endocardial Brg1 represses ADAMTS1 to maintain the microenvironment for myocardial morphogenesis. *Dev. Cell* 14 298–311. 10.1016/j.devcel.2007.11.018 18267097 PMC2274005

[B98] Sun H. Yang X. Zhu J. Lv T. Chen Y. Chen G. (2010). Inhibition of p300-HAT results in a reduced histone acetylation and down-regulation of gene expression in cardiac myocytes. *Life Sci.* 87 707–714. 10.1016/j.lfs.2010.10.009 21034749

[B99] Sun X. Hota S. K. Zhou Y. Q. Novak S. Miguel-Perez D. Christodoulou D. (2018). Cardiac-enriched BAF chromatin-remodeling complex subunit Baf60c regulates gene expression programs essential for heart development and function. *Biol. Open* 7:29512. 10.1242/bio.029512 29183906 PMC5829499

[B100] Sylva M. van den Hoff M. J. Moorman A. F. (2014). Development of the human heart. *Am. J. Med. Genet. A* 164A 1347–1371. 10.1002/ajmg.a.35896 23633400

[B101] Takaya T. Kawamura T. Morimoto T. Ono K. Kita T. Shimatsu A. (2008). Identification of p300-targeted acetylated residues in GATA4 during hypertrophic responses in cardiac myocytes. *J. Biol. Chem.* 283 9828–9835. 10.1074/jbc.M707391200 18252717

[B102] Takeuchi J. K. Bruneau B. G. (2009). Directed transdifferentiation of mouse mesoderm to heart tissue by defined factors. *Nature* 459 708–711. 10.1038/nature08039 19396158 PMC2728356

[B103] Trivedi C. M. Zhu W. Wang Q. Jia C. Kee H. J. Li L. (2010). Hopx and Hdac2 interact to modulate Gata4 acetylation and embryonic cardiac myocyte proliferation. *Dev. Cell* 19 450–459. 10.1016/j.devcel.2010.08.012 20833366 PMC2947937

[B104] Ulitsky I. Bartel D. P. (2013). lincRNAs: genomics, evolution, and mechanisms. *Cell* 154 26–46. 10.1016/j.cell.2013.06.020 23827673 PMC3924787

[B105] Vilardell M. Rasche A. Thormann A. Maschke-Dutz E. Perez-Jurado L. A. Lehrach H. (2011). Meta-analysis of heterogeneous Down Syndrome data reveals consistent genome-wide dosage effects related to neurological processes. *BMC Genomics* 12:229. 10.1186/1471-2164-12-229 21569303 PMC3110572

[B106] Vissers L. E. van Ravenswaaij C. M. Admiraal R. Hurst J. A. de Vries B. B. Janssen I. M. (2004). Mutations in a new member of the chromodomain gene family cause CHARGE syndrome. *Nat. Genet.* 36 955–957. 10.1038/ng1407 15300250

[B107] Voss T. C. Hager G. L. (2014). Dynamic regulation of transcriptional states by chromatin and transcription factors. *Nat. Rev. Genet.* 15 69–81. 10.1038/nrg3623 24342920 PMC6322398

[B108] Wang S. Su J. H. Beliveau B. J. Bintu B. Moffitt J. R. Wu C. T. (2016). Spatial organization of chromatin domains and compartments in single chromosomes. *Science* 353 598–602. 10.1126/science.aaf8084 27445307 PMC4991974

[B109] Wu B. Wang Y. Lui W. Langworthy M. Tompkins K. L. Hatzopoulos A. K. (2011). Nfatc1 coordinates valve endocardial cell lineage development required for heart valve formation. *Circ. Res.* 109 183–192. 10.1161/CIRCRESAHA.111.245035 21597012 PMC3132827

[B110] Yao Q. Chen Y. Zhou X. (2019). The roles of microRNAs in epigenetic regulation. *Curr. Opin. Chem. Biol.* 51 11–17. 10.1016/j.cbpa.2019.01.024 30825741

[B111] Yu M. Ren B. (2017). The Three-Dimensional Organization of Mammalian Genomes. *Annu. Rev. Cell Dev. Biol.* 33 265–289. 10.1146/annurev-cellbio-100616-060531 28783961 PMC5837811

[B112] Zhang W. Song M. Qu J. Liu G. H. (2018). Epigenetic Modifications in Cardiovascular Aging and Diseases. *Circ. Res.* 123 773–786. 10.1161/CIRCRESAHA.118.312497 30355081

[B113] Zoghbi H. Y. Beaudet A. L. (2016). Epigenetics and Human Disease. *Cold Spring Harb. Perspect. Biol.* 8:a019497. 10.1101/cshperspect.a019497 26834142 PMC4743078

[B114] Zufferey M. Tavernari D. Oricchio E. Ciriello G. (2018). Comparison of computational methods for the identification of topologically associating domains. *Genome Biol.* 19:217. 10.1186/s13059-018-1596-9 30526631 PMC6288901

